# Guy's Hospital Appeal and Its Basis

**Published:** 1913-05-31

**Authors:** 


### GUY'S HOSPITAL APPEAL AND ITS BASIS.

### What the Completion of the New Buildings Means.

On Tuesday next, June 3, Mr. A. J. Balfour comes to Guy's Hospital to open the new buildings of its medical and dental schools, which have at length been completed at a cost of £100,000, of which more than three-quarters have been subscribed. The occasion is one of the greatest importance to the public who have very little idea of what a medical school does for a hospital, and through the hospitals for the benefit of themselves. The present appeal is, therefore, an occasion for explaining what is the relation of a medical school to the public, and on what grounds it may appeal to them for support. The present appeal is the first appeal made by a medical school to the public. It is also as much a demonstration of the work actually accomplished as an appeal for funds to carry out extended activities. It marks the end of a period of work as much as the beginning of a new departure. As an instance of the value of a medical school to the public it is permissible to recall the responsibility recently thrown on Mr. Arbuthnot Lane, a Guy's man, who was selected to perform an operation on the Duchess of Connaught as the man uniquely capable of doing the particular piece of work required, whose natural abilities were trained by means of the opportunities afforded by the medical school attached to Guy's Hospital. It is unnecessary to multiply instances of the way in which the medical schools connected with the great hospitals provide the trained men to whom the public look in times of national or personal emergency caused by disease. In order that the public may have the best men and medical service possible it is absolutely essential that the medical schools should be organised in the best manner, and this can only be done by the continued specialisation of the varied departments, that is to say, by the constant recurring of fresh expense. What are the main departments of the medical and dental schools at Guy's? They consist of a department of physiology, a library, a museum, an elaborate dental school, besides anatomical, pathological, chemistry, and physics departments. All these have been built or rebuilt since 1897, with the result that the medical school at Guy's now claims to be the largest and most elaborately equipped in the United Kingdom.

The vast expenditure involved in this outlay has been provided by the generosity and personal sacrifice of the hospital's governors and the medical staff. The former have given largely and induced their friends to give. The latter have voluntarily foregone a large part of the income formerly paid to them from students' fees. It has been felt that students' fees should be used not to pay off the interest on a bank loan, but for the benefit of the students themselves. For it must be remembered that, while Guy's seems to have an entry more stable than some other schools have been able to maintain, the number of men entering the medical profession is falling seriously. Those who do enter are of the public-school class, a class, that is to say, which is unable in the mass to afford the expense of a long technical course without the assistance offered by a liberal supply of scholarships and exhibitions. The connection between the public schools and the medical schools may well be a close one, and it is interesting to note that many of Mr. Balfour's hearers on Tuesday will be schoolmasters.

What, then, is the present position? It is this. The two schools, medical and dental, have been built. It now remains to endow them. The scaffolding of the future has been constructed. But a school is more than a collection of laboratories and class-rooms. It is the corporate life of students and teachers through the medium of these, and under the inspiration of scholastic prizes and special researches which the endowment of exhibitions and scholarships, both special and general, alone make possible.

It is a curious and lamentable fact that the British public is as slow to endow education as it is quick to endow curative treatment. It is Mr. Carnegie, an American, who endows education, and he brought the fashion from the States and has not found many imitators in this country. In particular has this been the case with the medical schools. They have hitherto attracted few, if any, really large gifts, although without them there would be no doctors. Indeed so true is this, that the medical schools will have to go under if some help is not given to them. Amalgamation has already been talked of, and, within limits, has arguments which recommend its adoption, but which do not affect a medical school like Guy's which has already so often proved a pioneer. Its boast is to have been the first medical school to found a dental school for its students; the first to start a residential college; the first to amalgamate the students' clubs—enterprises the success of which has attracted many imitators. It must be apparent from this that the school has done its part nobly and doggedly. It has provided the active work and the buildings. It turns naturally to the public for the endowment which will consolidate and extend their usefulness. For though much has been done, it is certain that the need for further extension and specialisation is not likely to grow less. The sum of £40,000 must be found to put the school upon a self-supporting basis.

The work of investigating the causes of disease and of devising new methods of treatment requires at least such a sum if the new schools are to continue giving, as they have hitherto done, the best work to the hospital, and through them to the public at large. As King Edward said, when as Prince of Wales he visited the school at the beginning of the rebuilding: "Once men of wealth and philanthropic aspirations realise that money given for the purposes of medical education directly benefits humanity, I cannot doubt that the spirit which has prompted the British public to provide by voluntary effort what in other countries is provided by the State, will prove effective in the present need."

